# Crosstalk Between MicroRNAs and Circular RNAs in Human Diseases: A Bibliographic Study

**DOI:** 10.3389/fcell.2021.754880

**Published:** 2021-10-18

**Authors:** Yu-Meng Chen, Yi-Li Zheng, Xuan Su, Xue-Qiang Wang

**Affiliations:** ^1^Department of Sport Rehabilitation, Shanghai University of Sport, Shanghai, China; ^2^Shanghai Shangti Orthopaedic Hospital, Shanghai, China

**Keywords:** microRNA, circular RNA, crosstalk, bibliography, cancer

## Abstract

**Background:** Crosstalk of circular RNAs (circRNAs) and microRNAs (miRNAs) refers to the communication and co-regulation between them. circRNAs can act as miRNAs sponges, and miRNAs can mediate circRNAs. They interact to regulate gene expression and participate in the occurrence and development of various human diseases.

**Methods:** Publications on the crosstalk between miRNAs and circRNAs in human diseases were collected from Web of Science. The collected material was limited to English articles and reviews. CiteSpace and Microsoft Excel were used for bibliographic analysis.

**Results:** A total of 1,013 papers satisfied the inclusion criteria. The publication outputs and types of researched diseases were analyzed, and bibliographic analysis was used to characterize the most active journals, countries, institutions, keywords, and references. The annual number of publications remarkably increased from 2011 to 2020. Neoplasm was the main research hotspot (*n* = 750 publications), and *Biochemical and Biophysical Research Communications* published the largest number of papers (*n* = 64) on this topic. Nanjing Medical University ranked first among institutions actively engaged in this field by publishing 72 papers, and China contributed 96.84% of the 1,013 papers (*n* = 981 publications) analyzed. Burst keywords in recent years included glioblastoma, miR-7, skeletal muscle, and non-coding RNA.

**Conclusion:** Crosstalk between miRNAs and circRNAs in human diseases is a popular research topic. This study provides important clues on research trends and frontiers.

## Introduction

MicroRNAs (miRNAs) are endogenous non-coding RNAs (ncRNAs) measuring ∼22 nucleotides in length; they were first discovered in 1993 (Lee et al., 1993; Bartel, 2004). Lee et al. (1993) reported that lin-4 negatively regulates the expression of lin-14 protein by binding to the 3′-untranslated region (3′UTR) of lin-14 messenger RNA (mRNA) in *Caenorhabditis elegans*. miRNAs are crucial post-transcriptional regulators of gene expression and known to pair with the 3′UTR of target mRNAs to either induce mRNA degradation or inhibit translation (Bartel, 2009). Moreover, these RNAs have been reported to be involved in the processes of various diseases, such as cancer (Iacona and Lutz, 2019; Iqbal et al., 2019), chronic pain (Lopez-Gonzalez et al., 2017; Tramullas et al., 2018), and cardiac diseases (Colpaert and Calore, 2019; Fitzsimons et al., 2020).

Circular RNAs (circRNAs) are endogenous ncRNAs with covalent closed-loop structures that were first discovered in viral RNA by Sanger et al. (1976). circRNAs possess high stability on account of their unique structure, which protects them from degradation by exonucleases (Jeck et al., 2013). This type of RNA has been confirmed to modulate gene expression by acting as miRNA sponges (Hansen et al., 2013a; Memczak et al., 2013) or RNA binding protein regulators (Li et al., 2015). Similar to miRNAs, circRNAs are involved in a number of human diseases, such as cancer (Wang et al., 2017; Patop and Kadener, 2018), neurodegenerative disorders (Floris et al., 2017; Huang et al., 2020), and cardiovascular diseases (Aufiero et al., 2019; Zhang et al., 2020).

Crosstalk of circRNAs and miRNAs refers to the communication and co-regulation between them. As two important gene-expression regulators, circRNAs interact with miRNAs and participate in regulating mRNA expression in various human diseases (Hansen et al., 2013a; Kristensen et al., 2019). Crosstalk between miRNAs and circRNAs has been extensively studied, but the relevant mechanisms remain incompletely understood. To date, two mechanisms have been identified: (1) circRNAs act as miRNA inhibitors/sponges. ciRS-7 (circular RNA sponge for miR-7), one of the most typical circRNAs, has been observed to show overlapping co-expression with miR-7 in mouse brain. ciRS-7 can sponge miR-7 to inhibit miR-7 activity extensively, thereby increasing the levels of miR-7 target genes (Hansen et al., 2013a). (2) miRNAs mediate circRNAs. ciRS-7, also known as CDR1as, has been confirmed to be cleaved by Argonaute 2 via miR-671 mediation in cultured cells (Hansen et al., 2011) and mouse brain (Kleaveland et al., 2018). Furthermore, ciRS-7 and its crosstalk with miRNAs have been found to exert a significant effect on sensorimotor gating and synaptic transmission (Piwecka et al., 2017).

Crosstalk between circRNAs and miRNAs may provide new avenues through which the mechanisms, diagnosis, and treatment of human diseases could be explored. Thus, in the present work, we provide a bibliographic analysis of the related literature to identify current global research trends and emerging topics in this field.

## Materials and Methods

### Data Acquisition and Inclusion Criteria

This bibliographic analysis included published articles and reviews that related to research on the crosstalk between circRNAs and miRNAs, and these publications focused on the occurrence and development of human diseases or might be beneficial for studying human diseases. Data were extracted from Science Citation Index Expanded (SCI-Expanded) of Web of Science (WoS) in March 2021. Searches were conducted using the following keywords: TI = (circRNA^∗^ or “circ RNA^∗^” OR “circular RNA^∗^” OR circularRNA^∗^) AND TI = (microRNA^∗^ OR mir^∗^ OR miRNA^∗^ OR “micro RNA^∗^” OR “small non-coding RNAs” OR “small non-coding RNA” OR “small RNA^∗^”).

Supplementary Figure 1 shows the process of literature selection. Language was restricted to English, and no species limitation was implemented. We selected articles and reviews, and excluded all other publication types, such as meeting abstracts and corrections. Studies focusing on human diseases were included, while research on other subjects, such as plants, were excluded. The search and screening process was performed by two authors; in the event of disagreement, the final decision was made by the corresponding author.

### Analytical Tools and Key Indicators

Microsoft Excel 2019 and CiteSpace V (version: 5.6.R5; Drexel University) were used for bibliographic analysis, and SPSS 22.0 (SPSS Inc., Chicago, IL, United States) was used for statistical analysis. CiteSpace is often used for bibliographic analysis with data derived from WoS (Liu et al., 2019; Xu and Sun, 2020; Yan et al., 2020). International Classification of Diseases 11th Revision (ICD-11) was used to classify the diseases evaluated in the 1,013 papers we obtained (World Health Organization [WHO], 2021).

The number of publications and citations, journal impact factor (JIF, 2019), h-index, category and JIF quartile were collected from WoS. The productivity of an individual or group was reflected by the number of articles published. The number of citations, IF and h-index were collected to assess the impact of individuals or groups on publications in this filed. IF values were acquired from Journal Citation Reports (2021), which is a highly suitable index for evaluating the influence of journals (Garfield, 2006). The h-index characterizes the scientific output of a researcher. If the h-index of a researcher is *h*, the researcher published at least *h* papers and each of these papers was cited at least *h* times (Hirsch, 2005). Each journal was assigned to at least one category in descending order according to JIF, and the top 25% of JIF distribution were Q1, 25–50% were Q2 and 50–75% were Q3. Centrality is an indicator calculated by CiteSpace to evaluate the importance of nodes in a network; nodes with high centrality indicate pivotal points (Chen, 2006).

## Results

### Publication Output

**FIGURE 1:**
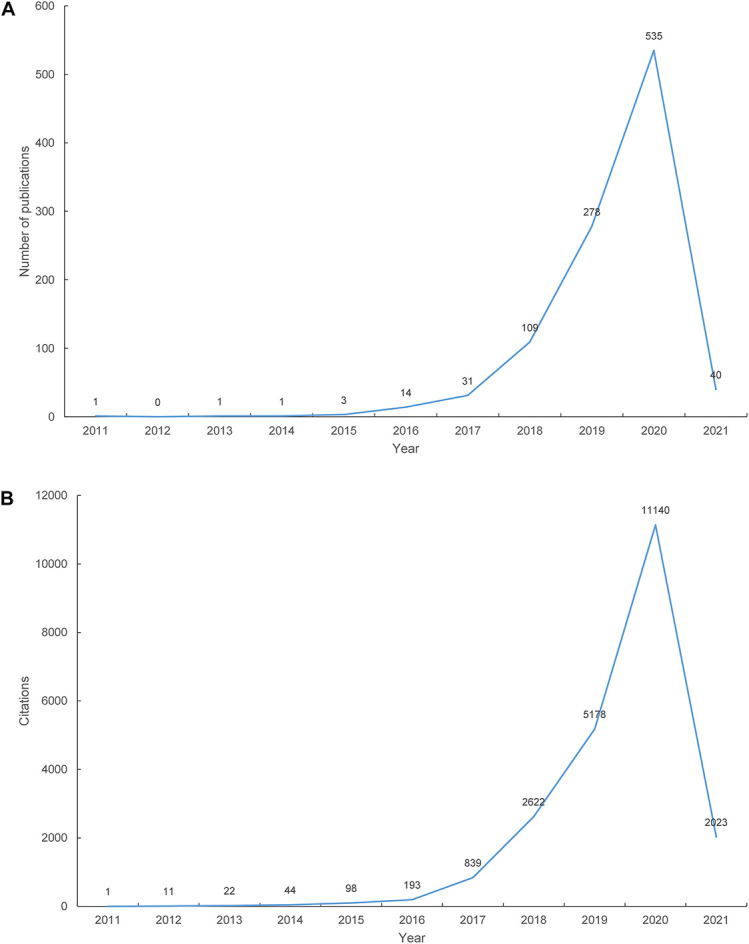
The number of publications and citations. **(A)** The number of annual publications on the crosstalk between microRNAs and circular RNAs from 2011 to 2021; **(B)** The number of annual citations on the crosstalk between microRNAs and circular RNAs from 2011 to 2021.

A total of 1,013 papers were included in our study. Figure 1A shows the number of articles published by year. The first paper related to the crosstalk between miRNAs and circRNAs in human diseases was published in 2011. The number of publications increased from 1 in 2011 to 535 in 2020. As of February 28, 2021, 40 papers on this topic have been published in 2021. Figure 1B shows the total number of citations of the 1,013 papers per year. Linear regression analysis showed that the number of publications (*t* = 3.376, *P* = 0.01 < 0.05, data of 2021 were not included because they are incomplete) and citations (*t* = 3.413, *P* = 0.009 < 0.05, data of 2021 were not included) increased remarkably each year from 2011 to 2020.

### Distribution of Diseases

The research directions of the 1,013 papers were classified and summarized according to ICD-11. Table 1 shows the top 10 diseases studied. Neoplasm was the most popular research topic related to the crosstalk between miRNAs and circRNAs (*n* = 750 publications; 74.04% of the total number of publications) and had an h-index of 56; this topic was followed by diseases of the circulatory system (*n* = 62 publications; h-index = 17) and musculoskeletal system or connective tissue (*n* = 57 publications; h-index = 15).

**TABLE 1:** The top 10 diseases ranked by number of publications.

**Rank**	**Disease**	**Publication**	**Citations**	**H-index**
1	Neoplasms	750	15435	56
2	Diseases of the circulatory system	62	1756	17
3	Diseases of the musculoskeletal system or connective tissue	57	1031	15
4	Diseases of the nervous system	21	557	10
5	Diseases of the visual system	12	120	5
6	Diseases of the respiratory system	12	56	4
7	Diseases of the digestive system	11	141	4
8	Certain infectious or parasitic diseases	10	78	4
9	Endocrine, nutritional or metabolic diseases	10	425	5
10	Symptoms, signs or clinical findings, not elsewhere classified	10	64	3

### Distribution by Journals

A total of 244 journals contributed the 1,013 papers included in this study. Among the top 10 journals ranked by number of publications (Table 2), *Biochemical and Biophysical Research Communications* contributed the greatest number of publications (*n* = 64) and had the highest h-index (33), followed by *European Review for Medical and Pharmacological Sciences* (*n* = 43 publications) and *Journal of Cellular Biochemistry* (*n* = 38 publications). The IF of the top 10 journals ranged from 2.886 to 15.302 (mean = 5.4113). Among the journals contributing publications on the crosstalk between miRNAs and circRNAs in human diseases, *Molecular Cancer* (*n* = 26 publications) had the highest IF (IF 2019 = 15.302), followed by *Molecular Therapy-Nucleic Acids* (*n* = 28 publications; IF 2019 = 7.032) and *Cell Death and Disease* (*n* = 25 publications; IF 2019 = 6.304). Among the top 10 journals, 33.33% JIF quartile were Q1, 46.67% were Q2 and 20% were Q3.

**TABLE 2:** The top 10 journals ranked by number of publications.

**Rank**	**Journal**	**Publication**	**H-index**	**JIF (2019)**	**Category (Web of Science)**	**JIF Quartile (2019)**
1	Biochemical and Biophysical Research Communications	64	33	2.985	Biochemistry and molecular biology biophysics	Q3; Q2
2	European Review for Medical and Pharmacological Sciences	43	8	3.024	Pharmacology and pharmacy	Q2
3	Journal of Cellular Biochemistry	38	13	4.237	Cell biology biochemistry and molecular biology	Q2; Q2
4	Oncotargets and Therapy	35	6	3.337	Oncology biotechnology and applied microbiology	Q3; Q2
5	Cancer Cell International	33	8	4.175	Oncology	Q2
6	Aging-US	32	10	4.831	Geriatrics and gerontology cell biology	Q1; Q2
7	Cancer Management and Research	32	8	2.886	Oncology	Q3
8	Molecular Therapy-Nucleic Acids	28	12	7.032	Medicine, research and experimental	Q1
9	Molecular Cancer	26	18	15.302	Oncology biochemistry and molecular biology	Q1; Q1
10	Cell Death and Disease	25	10	6.304	Cell biology	Q1

*JIF, journal impact factor.*

**FIGURE 2:**
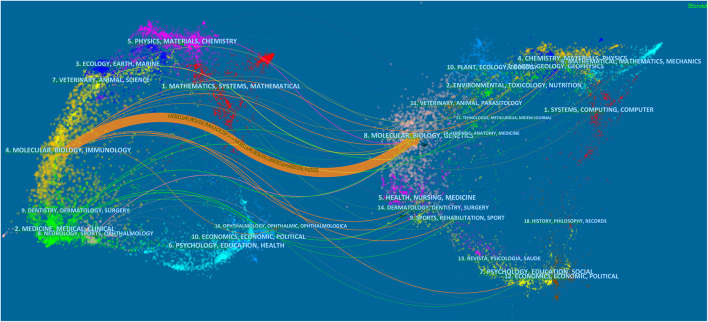
The dual-map overlay of journals that published articles on the crosstalk between microRNAs and circular RNAs.

A dual map of journals presenting contributions and connections between disciplines is shown in Figure 2. The left-hand side of the map presents citing journals, whereas the right-hand side presents cited journals. The 1,013 papers obtained from our database search were mostly published in journals dedicated to molecular, biology and immunology field, and cited journal publications on molecular, biology, and genetics field.

### Distribution by Countries and Institutions

**FIGURE 3:**
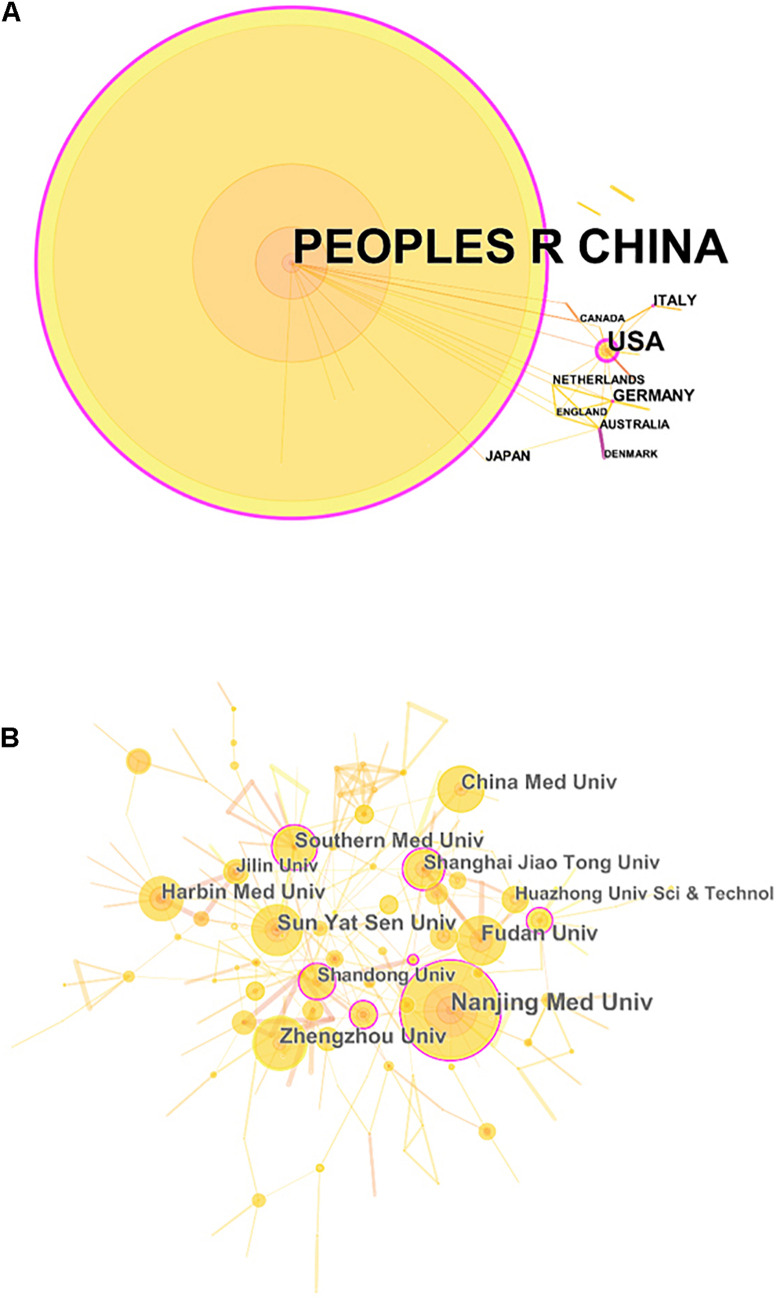
The analysis of countries/regions and institutions. **(A)** Network map of countries/regions engaged in research on the crosstalk between microRNAs and circular RNAs; **(B)** Network map of institutions engaged in research on the crosstalk between microRNAs and circular RNAs.

**FIGURE 4:**
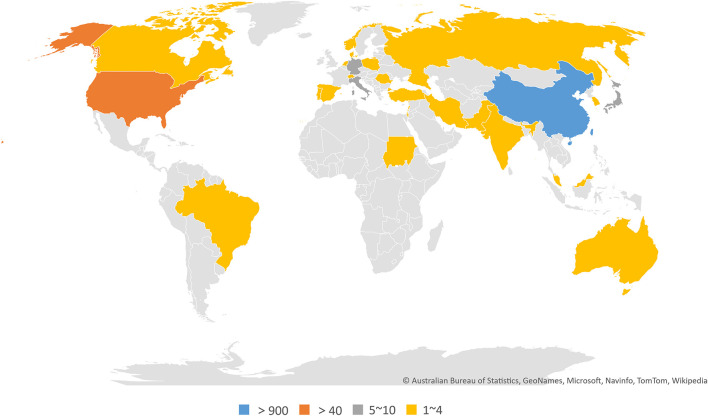
World map of countries/regions that published articles on the crosstalk between microRNAs and circular RNAs.

In total, 30 countries and 748 institutions contributed to the related research, and the details of each item can be found in Supplementary Tables 1, 2. Figures 3A,B, respectively, show the contributions and collaborations by country and by institution. The distribution of countries is presented in Figure 4. Table 3 shows the top 10 countries ranked by distribution of publications. China contributed 96.84% of the 1,013 papers (*n* = 981 publications), with a high centrality of 0.57. The United States revealed the highest centrality (0.59) and contributed the second highest number of publications (*n* = 42).

**TABLE 3:** The top 10 countries ranked by number of publications.

**Rank**	**Country**	**Publication**	**Centrality**
1	China*	981	0.57
2	United States	42	0.59
3	Germany	8	0.10
4	Italy	6	0.10
5	Japan	5	0.00
6	Australia	4	0.13
7	Netherlands	4	0.00
8	Canada	3	0.04
9	Denmark	3	0.00
10	England	3	0.00

*Includes the data of Taiwan (5 publications).

The top 10 institutions ranked by number of publications were all universities in China (Table 4). Among these institutions, Nanjing Medical University ranked first (*n* = 72 publications), followed by Zhengzhou University (*n* = 43 publications) and Sun Yat-sen University (*n* = 41 publications).

**TABLE 4:** The top 10 institutions ranked by number of publications.

**Rank**	**Institution**	**Publication**	**Country**
1	Nanjing Medical University	72	China
2	Zhengzhou University	43	China
3	Sun Yat-sen University	41	China
4	Fudan University	40	China
5	China Medical University	37	China
6	Harbin Medical University	36	China
7	Southern Medical University	34	China
8	Shanghai Jiao Tong University	32	China
9	Shandong University	30	China
10	Huazhong University of Science and Technology	24	China

### Analysis of Keywords

**FIGURE 5:**
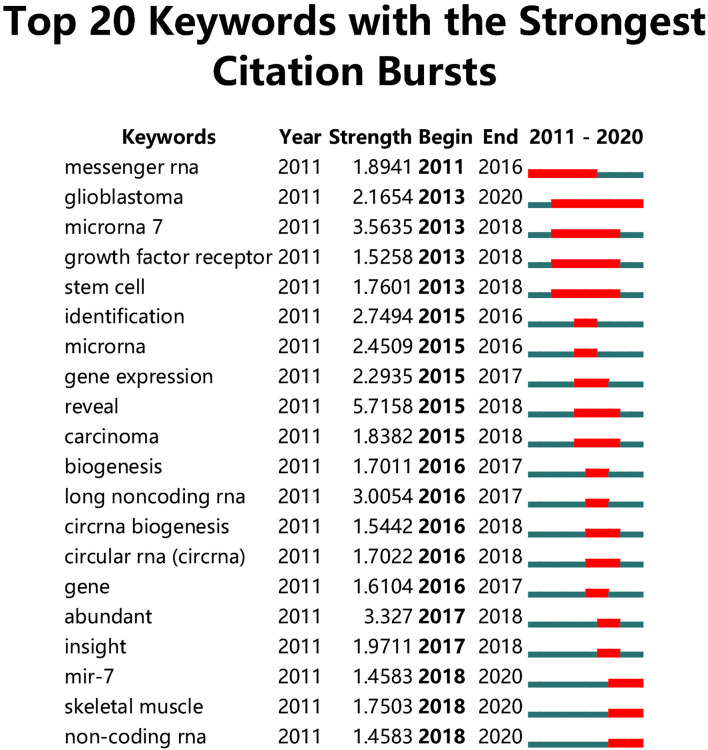
The top 20 keywords with the strongest citation bursts of publications on the crosstalk between microRNAs and circular RNAs.

The top 20 keywords with the strongest citation bursts at different time periods are shown in Figure 5. Messenger RNA was the first keyword with the strongest citation burst, which began in 2011 and ended in 2016. Glioblastoma, mir-7, skeletal muscle, and ncRNA were keywords with strong citation bursts that lasted until 2020. Among these four keywords, glioblastoma revealed the highest strength (3.5635) and was the first to burst (in 2013).

### Analysis of References

**FIGURE 6:**
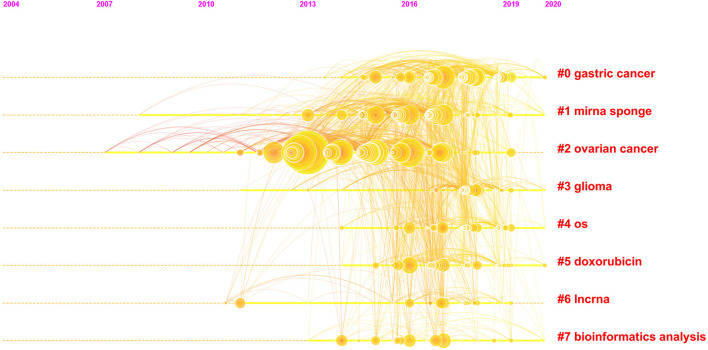
The analysis of references. Co-citation map (timeline view) of references from publications on the crosstalk between microRNAs and circular RNAs.

References are an indispensable part of publications. Figure 6 illustrates a timeline view of references in the 1,013 publications related to the crosstalk between microRNAs and circRNAs in human diseases. The timeline view was constructed using CiteSpace, and the cluster labels were extracted from the keywords. Figure 6 shows the top 8 clusters, the first of which is marked #0. Gastric cancer revealed the largest cluster, followed by miRNA sponge (#1), ovarian cancer (#2), and glioma (#3).

## Discussion

In this study, we performed a bibliographic analysis of studies on the crosstalk between miRNAs and circRNAs and found that research on this topic is well underway. circRNAs can communicate and co-regulate with miRNAs to regulate gene expression. Hansen and his colleagues published the first paper on the crosstalk between ciRS-7 and miR-671 in 2011 (Hansen et al., 2011). However, the number of publications on this subject exceed 10 per year until 2016. Thereafter, publications exceeded 100 papers per year in 2018 and reached 535 papers per year in 2020.

The mechanisms of crosstalk between miRNAs and circRNAs are incompletely understood. miRNAs and circRNAs have been proven to interact in the pathological process of many human diseases, including cancer, osteoarthritis (OA) and neuropathic pain (NP). circRNAs sponging miRNAs was a frequently mentioned mechanism of the crosstalk between circRNAs and miRNAs in human diseases. Zheng et al. (2016) revealed that circHIPK3, a circRNAs differently expresses between cancer and normal tissues, has 18 potential binding sites and can sponge 9 miRNAs to modulate human cell proliferation. Liu et al. (2016) reported that circRNA-CER competitively binds miR-136 to regulated MMP13 expression and participated in the degradation of chondrocyte extracellular matrix. Several circRNAs have been confirmed to regulate mRNA expression by sponge miRNAs, and then involved in neuroinflammation, neuronal autophagy, cell proliferation, and central sensitization in NP (Song et al., 2020). About the mechanisms of cancer, Hanse found miR-671 can directionally cleave ciRS-7 to release miR-7 that sponged by ciRS-7 (Hansen et al., 2013b). However, few studies researched the mechanism of miRNAs mediating circRNAs.

Neoplasms are the most common condition associated with research on the crosstalk between miRNAs and circRNAs; indeed, articles discussing neoplasms made up 74.04% of the total number of publications obtained, with a high h-index (56). Han and his colleagues revealed that circMTO1 inhibits the process of hepatocellular carcinoma by sponging miR-9 (Han et al., 2017). Zhang and his colleagues found that circFGFR1 upregulates the expression of CXCR4, the miR-381-3p target gene, to promote the progression of non-small cell lung cancer by miR-381-3p (Zhang et al., 2019b). The research findings of Zhang et al. (2017) were published in *Molecular Cancer*, which had the highest IF among the top 10 journals ranked by number of publications. Interactions between miRNAs and circRNAs have also been explored in other types of neoplasms, such as gastric cancer, breast cancer (Yang et al., 2019) and glioma (Xu et al., 2018). Most of the related research focuses on malignant tumors and the mechanism of circRNA as a miRNA sponge.

*Biochemical and Biophysical Research Communications* contributed the most to research on the crosstalk between miRNAs and circRNAs by publishing 64 articles with the highest h-index (33). However, in terms of IF, this journal placed ninth among the top 10 journals ranked by number of publications. *Molecular Cancer*, the IF of which was 15.302, was the only journal with an IF greater than 10 among the top 10 journals. Although the IF of the top 10 journals was not high, JIF Quartile shown that these journals ranked well in corresponding categories (33.33% of Q1 and 46.67% of Q2). These results suggest that the quality of most publications was reliable, but more high quality and ground-breaking researches are needed in this field. Furthermore, the crosstalk between circRNAs and miRNAs is a relatively new field, and it may be a possible reason for these results.

China is the major country contributing research on the crosstalk between miRNAs and circRNAs in human diseases. The country published 981 papers (96.84% of the total number of articles included in this work) and showed fairly high centrality (0.57). The United States ranked second in terms of countries with the greatest number of publications; the country published 42 papers and demonstrated the highest centrality (0.59) among the countries compared. Interestingly, China noticeably contributed more publications than the United States, but the centrality of the former was lower than that of the latter. This finding may be explained by the vast number of articles published by China alone, which could result in low centrality. The top 10 institutions ranked by number of publications were all Chinese universities, among which Nanjing Medical University ranked first, with 72 publications. Therefore, China is the leading country in research on the crosstalk between miRNAs and circRNAs in human diseases, and universities are the main institutional form in this field.

According to CiteSpace V, “glioblastoma,” “miR-7,” “skeletal muscle,” and “non-coding RNA” are the most popular keywords in the related research; these terms indicate potential research hotspots and frontiers. The four potential frontiers of research on the crosstalk between miRNAs and circRNAs in human diseases are as follows:

(1) Glioblastoma: Glioblastoma multiforme (GBM), the most common type of brain cancer, is also known as grade IV glioma; the disease has a short overall survival time and high malignancy (Westphal and Lamszus, 2011; Louis et al., 2016). Although therapeutic strategies for this disease have been improved, GBM remains difficult to treat (Omuro and DeAngelis, 2013). circRNAs and miRNAs play important roles in many malignancies, and their crosstalk in GBM remains unclear. Thus, research on this field has great potential therapeutic significance.
(2) MiR-7: miR-7 can modulate the expression of several oncogenes, and changes in miR-7 activity can affect the progression of cancer (Hansen et al., 2013b). The mechanism underlying the crosstalk between circRNAs and miR-7 in human diseases is a research hotspot.
(3) Skeletal muscle: Skeletal muscle, which contains 50–75% of all proteins in the human body, accounts for approximately 40% of the human body weight. Skeletal muscle is closely related to human locomotion and metabolism, and skeletal muscle diseases can severely affect a patient’s quality of life (Frontera and Ochala, 2015). circRNAs and miRNAs can regulate the development of skeletal muscle, and the crosstalk between these RNAs is a research frontier (Ge and Chen, 2011; Zhang et al., 2019a).
(4) Non-coding RNA: Research conducted over the last decade has reported that ncRNAs are involved in several physiological and pathological processes. circRNAs and miRNAs are two types of ncRNAs that do not code proteins but can regulate gene expression. The effect of crosstalk between circRNAs and miRNAs on the complex and unknown regulatory networks of ncRNA must be further investigated (Panni et al., 2020).

According to our analysis of keywords and references, research on the crosstalk between circRNAs and miRNAs mainly focuses on cancer, including glioma, gastric cancer, and ovarian cancer, and the related sponge mechanism. Journals in molecular, biology and genetics field were the main source of references for the published research. Most publications were published in journals dedicated to molecular, biology and immunology field.

To the best of our knowledge, this study is the first to use CiteSpace to perform a bibliographic analysis of publications on the crosstalk between miRNAs and circRNAs. However, this study presents some limitations. First, SCI-E of WoS, although an authoritative and comprehensive database in the medical field, was the only resource we selected for data acquisition; thus, some important findings published in other databases may have been missed. Second, the contributions and collaborations of authors were not analyzed because most of the authors were from China and different Chinese names could be translated to the same English name. Third, this study lacked an assessment of the overall quality of publications.

## Conclusion

A total of 1,013 papers on the crosstalk between miRNAs and circRNAs in human diseases were published. The first paper was published in 2011 and initiated research in this field. The quality of most publications was relatively well in this field, but high-impact researches were needed. *Biochemical and Biophysical Research Communications* contributed the most to this field in terms of number of publications, and China produced the largest amount of research on this topic. In terms of institution, Nanjing Medical University published the largest number of related articles. At present, cancer is the most popular research area related to the crosstalk between miRNAs and circRNAs in human diseases. The latest burst keywords are “glioblastoma,” “miR-7,” “skeletal muscle,” and “non-coding RNA.” As far as we know, no bibliographic analysis has been conducted for publications on the crosstalk between circRNAs and miRNAs before. This study may provide researchers important clues on research trends and frontiers in this field.

## Data Availability Statement

The original contributions presented in the study are included in the article/Supplementary Material, further inquiries can be directed to the corresponding author.

## Author Contributions

Y-MC and X-QW: conceptualization and visualization. Y-MC: data curation, formal analysis, methodology, and writing – original draft. X-QW: funding acquisition and supervision. Y-MC, Y-LZ, XS, and X-QW: validation. Y-MC, Y-LZ, and XS: writing, review, and editing. All authors contributed to the article and approved the submitted version.

## Conflict of Interest

The authors declare that the research was conducted in the absence of any commercial or financial relationships that could be construed as a potential conflict of interest.

## Publisher’s Note

All claims expressed in this article are solely those of the authors and do not necessarily represent those of their affiliated organizations, or those of the publisher, the editors and the reviewers. Any product that may be evaluated in this article, or claim that may be made by its manufacturer, is not guaranteed or endorsed by the publisher.

## Supplementary Material

Supplementary Figure 1 Flowchart of literature selection on the crosstalk between microRNAs and circular RNAs in human diseases.
