# Supplementary figures and images for: Crosstalk Between MicroRNAs and Circular RNAs in Human Diseases: A Bibliographic Study
Source: Front Cell Dev Biol. 2021 Oct 18;9:754880. doi: 10.3389/fcell.2021.754880 (PMC8558455; doi:10.3389/fcell.2021.754880)

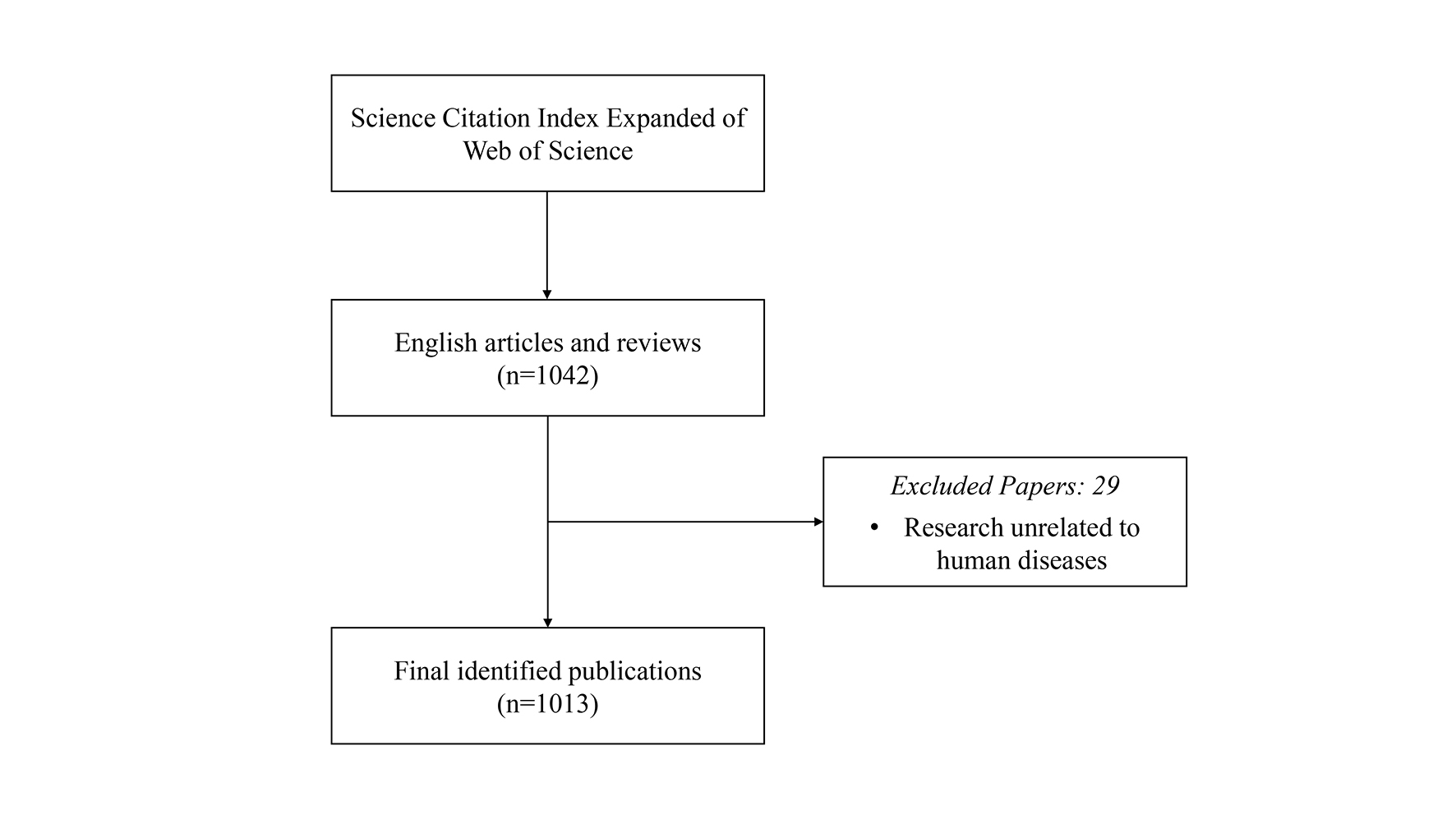

Supplement: Supplementary Figure 1 — Flowchart of literature selection on the crosstalk between microRNAs and circular RNAs in human diseases. [file Image_1.JPEG]
