# Supplementary material for: Crosstalk Between MicroRNAs and Circular RNAs in Human Diseases: A Bibliographic Study
Source: Front Cell Dev Biol. 2021 Oct 18;9:754880. doi: 10.3389/fcell.2021.754880 (PMC8558455; doi:10.3389/fcell.2021.754880)
Supplement: Supplementary file 2 [file Table_1.DOCX]

**Supplementary Table 1.** Raw data on countries of the publications on the crosstalk between microRNAs and circular RNAs in human diseases

| **Countries/Regions** | **Records** | **% of 1013** |
| --- | --- | --- |
| PEOPLES R CHINA | 976 | 96.347 |
| USA | 42 | 4.146 |
| GERMANY | 8 | 0.79 |
| ITALY | 6 | 0.592 |
| JAPAN | 5 | 0.494 |
| TAIWAN | 5 | 0.494 |
| AUSTRALIA | 4 | 0.395 |
| NETHERLANDS | 4 | 0.395 |
| CANADA | 3 | 0.296 |
| DENMARK | 3 | 0.296 |
| ENGLAND | 3 | 0.296 |
| BRAZIL | 2 | 0.197 |
| INDIA | 2 | 0.197 |
| POLAND | 2 | 0.197 |
| PORTUGAL | 2 | 0.197 |
| SINGAPORE | 2 | 0.197 |
| SOUTH KOREA | 2 | 0.197 |
| IRAN | 1 | 0.099 |
| ISRAEL | 1 | 0.099 |
| LEBANON | 1 | 0.099 |
| MALAYSIA | 1 | 0.099 |
| NORWAY | 1 | 0.099 |
| PAKISTAN | 1 | 0.099 |
| ROMANIA | 1 | 0.099 |
| RUSSIA | 1 | 0.099 |
| SCOTLAND | 1 | 0.099 |
| SPAIN | 1 | 0.099 |
| SUDAN | 1 | 0.099 |
| SWITZERLAND | 1 | 0.099 |
| TURKEY | 1 | 0.099 |
| WALES | 1 | 0.099 |
