# Supplementary material for: Crosstalk Between MicroRNAs and Circular RNAs in Human Diseases: A Bibliographic Study
Source: Front Cell Dev Biol. 2021 Oct 18;9:754880. doi: 10.3389/fcell.2021.754880 (PMC8558455; doi:10.3389/fcell.2021.754880)
Supplement: Supplementary file 3 [file Table_2.DOCX]

**Supplementary Table 2.** Raw data on institutions of the publications on the crosstalk between microRNAs and circular RNAs in human diseases

| **Institutions** | **Records** | **% of 1013** |
| --- | --- | --- |
| NANJING MED UNIV | 72 | 7.108 |
| ZHENGZHOU UNIV | 43 | 4.245 |
| SUN YAT SEN UNIV | 41 | 4.047 |
| FUDAN UNIV | 40 | 3.949 |
| CHINA MED UNIV | 37 | 3.653 |
| HARBIN MED UNIV | 36 | 3.554 |
| SOUTHERN MED UNIV | 34 | 3.356 |
| SHANGHAI JIAO TONG UNIV | 32 | 3.159 |
| SHANDONG UNIV | 30 | 2.962 |
| HUAZHONG UNIV SCI TECHNOL | 24 | 2.369 |
| JILIN UNIV | 23 | 2.27 |
| TONGJI UNIV | 22 | 2.172 |
| QINGDAO UNIV | 21 | 2.073 |
| SOOCHOW UNIV | 21 | 2.073 |
| QIQIHAR MED UNIV | 20 | 1.974 |
| XI AN JIAO TONG UNIV | 20 | 1.974 |
| XUZHOU MED UNIV | 20 | 1.974 |
| ANHUI MED UNIV | 18 | 1.777 |
| NANCHANG UNIV | 18 | 1.777 |
| CENT SOUTH UNIV | 17 | 1.678 |
| GUANGZHOU MED UNIV | 17 | 1.678 |
| CAPITAL MED UNIV | 16 | 1.579 |
| SECOND MIL MED UNIV | 16 | 1.579 |
| SOUTHEAST UNIV | 16 | 1.579 |
| WENZHOU MED UNIV | 16 | 1.579 |
| CENT S UNIV | 15 | 1.481 |
| HENAN UNIV | 15 | 1.481 |
| PEKING UNIV | 14 | 1.382 |
| FUJIAN MED UNIV | 13 | 1.283 |
| ZHEJIANG UNIV | 13 | 1.283 |
| CHONGQING MED UNIV | 12 | 1.185 |
| JINAN UNIV | 12 | 1.185 |
| BENGBU MED COLL | 10 | 0.987 |
| CHINESE ACAD SCI | 10 | 0.987 |
| NANTONG UNIV | 10 | 0.987 |
| SICHUAN UNIV | 10 | 0.987 |
| WUHAN UNIV | 10 | 0.987 |
| XIAMEN UNIV | 10 | 0.987 |
| CHINESE ACAD MED SCI | 9 | 0.888 |
| HEBEI MED UNIV | 9 | 0.888 |
| NANJING UNIV | 9 | 0.888 |
| CHINESE PEOPLES LIBERAT ARMY GEN HOSP | 8 | 0.79 |
| JIANGSU UNIV | 8 | 0.79 |
| JINING 1 PEOPLES HOSP | 8 | 0.79 |
| NANJING UNIV CHINESE MED | 8 | 0.79 |
| HEILONGJIANG PROV HOSP | 7 | 0.691 |
| KUNMING MED UNIV | 7 | 0.691 |
| SHANGHAI UNIV TRADIT CHINESE MED | 7 | 0.691 |
| WANNAN MED COLL | 7 | 0.691 |
| BINZHOU MED UNIV | 6 | 0.592 |
| HUBEI UNIV MED | 6 | 0.592 |
| JINING MED UNIV | 6 | 0.592 |
| LANZHOU UNIV | 6 | 0.592 |
| NORTHWEST A F UNIV | 6 | 0.592 |
| SECOND PEOPLES HOSP HUAIAN | 6 | 0.592 |
| SHANGHAI UNIV MED HLTH SCI | 6 | 0.592 |
| SHENZHEN UNIV | 6 | 0.592 |
| SOUTHWEST MED UNIV | 6 | 0.592 |
| UNIV CHINESE ACAD SCI | 6 | 0.592 |
| UNIV SCI TECHNOL CHINA | 6 | 0.592 |
| XINJIANG MED UNIV | 6 | 0.592 |
| GUANGXI MED UNIV | 5 | 0.494 |
| GUIZHOU MED UNIV | 5 | 0.494 |
| HANGZHOU MED COLL | 5 | 0.494 |
| HENAN UNIV SCI TECHNOL | 5 | 0.494 |
| JIANGNAN UNIV | 5 | 0.494 |
| JINZHOU MED UNIV | 5 | 0.494 |
| SHANDONG FIRST MED UNIV | 5 | 0.494 |
| WEIFANG PEOPLES HOSP | 5 | 0.494 |
| XIAN MED UNIV | 5 | 0.494 |
| XINXIANG MED UNIV | 5 | 0.494 |
| ZHEJIANG CHINESE MED UNIV | 5 | 0.494 |
| FIRST HOSP JILIN UNIV | 4 | 0.395 |
| FIRST HOSP QIQIHAR | 4 | 0.395 |
| GUANGDONG MED UNIV | 4 | 0.395 |
| HARVARD MED SCH | 4 | 0.395 |
| HENAN PROV PEOPLES HOSP | 4 | 0.395 |
| HEZE MUNICIPAL HOSP | 4 | 0.395 |
| JIANGSU CANC HOSP | 4 | 0.395 |
| PEKING UNION MED COLL | 4 | 0.395 |
| SECOND HOSP JILIN UNIV | 4 | 0.395 |
| SHANXI MED UNIV | 4 | 0.395 |
| TIANJIN MED UNIV GEN HOSP | 4 | 0.395 |
| UNIV ROCHESTER | 4 | 0.395 |
| WEIFANG MED UNIV | 4 | 0.395 |
| ZAOZHUANG MUNICIPAL HOSP | 4 | 0.395 |
| AARHUS UNIV | 3 | 0.296 |
| AIR FORCE MED UNIV | 3 | 0.296 |
| CHINA ACAD CHINESE MED SCI | 3 | 0.296 |
| CHONGQING UNIV | 3 | 0.296 |
| DALIAN MED UNIV | 3 | 0.296 |
| GANNAN MED UNIV | 3 | 0.296 |
| GANSU PROV HOSP | 3 | 0.296 |
| GUILIN MED UNIV | 3 | 0.296 |
| HEBEI PROV CANGZHOU HOSP INTEGRATED TRADIT WEST | 3 | 0.296 |
| HUAIAN SECOND PEOPLES HOSP | 3 | 0.296 |
| JIAMUSI UNIV | 3 | 0.296 |
| JIAXING UNIV | 3 | 0.296 |
| KEY LAB MUSCULOSKELETAL SYST DEGENERAT REGENERA | 3 | 0.296 |
| KUNMING UNIV SCI TECHNOL | 3 | 0.296 |
| LINYI CENT HOSP | 3 | 0.296 |
| LOMA LINDA UNIV | 3 | 0.296 |
| SHANDONG UNIV TRADIT CHINESE MED | 3 | 0.296 |
| SHANGHAI JIAO TONG UNIV AFFILIATED PEOPLES HOSP 6 | 3 | 0.296 |
| SHANTOU UNIV | 3 | 0.296 |
| SHANXI PROV PEOPLES HOSP | 3 | 0.296 |
| SHANXIAN CENT HOSP | 3 | 0.296 |
| SHENGLI OILFIELD CENT HOSP | 3 | 0.296 |
| TIANJIN MED UNIV | 3 | 0.296 |
| UNIV SOUTH CHINA | 3 | 0.296 |
| XUZHOU CENT HOSP | 3 | 0.296 |
| YANTAI YUHUANGDING HOSP | 3 | 0.296 |
| ZHEJIANG PROV PEOPLES HOSP | 3 | 0.296 |
| 181 HOSP PLA | 2 | 0.197 |
| ANHUI PROV HOSP | 2 | 0.197 |
| ARMY MED UNIV | 2 | 0.197 |
| BAYLOR COLL MED | 2 | 0.197 |
| CANGZHOU CENT HOSP | 2 | 0.197 |
| CENT HOSP PANYU DIST | 2 | 0.197 |
| CHENGDU MED COLL | 2 | 0.197 |
| CHENGDU UNIV TRADIT CHINESE MED | 2 | 0.197 |
| CHINESE ACAD AGR SCI | 2 | 0.197 |
| CHINESE ACAD MED SCI PEKING UNION MED COLL | 2 | 0.197 |
| CHINESE PEOPLES LIBERAT ARMY NAVY 971 HOSP | 2 | 0.197 |
| CNR | 2 | 0.197 |
| DALIAN UNIV | 2 | 0.197 |
| EASTERN HEPATOBILIARY SURG HOSP | 2 | 0.197 |
| FIRST HOSP HARBIN | 2 | 0.197 |
| FIRST PEOPLES HOSP LIANYUNGANG | 2 | 0.197 |
| FIRST PEOPLES HOSP TAIZHOU | 2 | 0.197 |
| FIRST PEOPLES HOSP WENLING | 2 | 0.197 |
| FIRST PEOPLES HOSP YIBIN | 2 | 0.197 |
| FIRST PEOPLES HOSP YUNNAN PROV | 2 | 0.197 |
| FOURTH MIL MED UNIV | 2 | 0.197 |
| FUJIAN CANC HOSP | 2 | 0.197 |
| FUJIAN PROV HOSP | 2 | 0.197 |
| GANZHOU PEOPLES HOSP | 2 | 0.197 |
| GERMAN CANC RES CTR | 2 | 0.197 |
| GUANGDONG ACAD MED SCI | 2 | 0.197 |
| GUANGDONG CARDIOVASC INST | 2 | 0.197 |
| GUANGDONG GEN HOSP | 2 | 0.197 |
| GUANGDONG PHARMACEUT UNIV | 2 | 0.197 |
| GUANGDONG PROV PEOPLES HOSP | 2 | 0.197 |
| GUANGZHOU UNIV CHINESE MED | 2 | 0.197 |
| GUIZHOU PROV PEOPLES HOSP | 2 | 0.197 |
| HARBIN MED UNIV DAQING | 2 | 0.197 |
| HEBEI ENGN UNIV | 2 | 0.197 |
| HENAN NORMAL UNIV | 2 | 0.197 |
| HOSP CHENGDU UNIV TRADIT CHINESE MED | 2 | 0.197 |
| HOWARD UNIV | 2 | 0.197 |
| HUBEI POLYTECH UNIV | 2 | 0.197 |
| HUNAN UNIV CHINESE MED | 2 | 0.197 |
| JIANGSU INST CANC RES | 2 | 0.197 |
| JIANGSU PROV HOSP | 2 | 0.197 |
| JIANGXI PROV CHILDRENS HOSP | 2 | 0.197 |
| JOHNS HOPKINS UNIV | 2 | 0.197 |
| KAIFENG CENT HOSP | 2 | 0.197 |
| KEY LAB BASIC RES HEAVY ION RADIAT APPLICAT MED | 2 | 0.197 |
| KUNMING CHILDRENS HOSP | 2 | 0.197 |
| LIAN SHUI PEOPLES HOSP | 2 | 0.197 |
| LIAOCHENG PEOPLES HOSP | 2 | 0.197 |
| LIAONING CLIN MED RES CTR NERVOUS SYST DIS | 2 | 0.197 |
| LINYI PEOPLES HOSP | 2 | 0.197 |
| LISHUI MUNICIPAL CENT HOSP | 2 | 0.197 |
| MEHARRY MED COLL | 2 | 0.197 |
| MIANYANG CENT HOSP | 2 | 0.197 |
| MINIST AGR | 2 | 0.197 |
| MINIST EDUC | 2 | 0.197 |
| MINIST HLTH | 2 | 0.197 |
| MUDANJIANG MED UNIV | 2 | 0.197 |
| NANCHONG CENT HOSP | 2 | 0.197 |
| NATL UNIV SINGAPORE | 2 | 0.197 |
| NAVY MED UNIV | 2 | 0.197 |
| NINGBO 2 HOSP | 2 | 0.197 |
| NINGBO UNIV | 2 | 0.197 |
| NINGXIA MED UNIV | 2 | 0.197 |
| PEOPLES HOSP LIAONING PROV | 2 | 0.197 |
| PEOPLES HOSP RIZHAO | 2 | 0.197 |
| QINGDAO 6 PEOPLES HOSP | 2 | 0.197 |
| QINGDAO MUNICIPAL HOSP | 2 | 0.197 |
| SECOND PEOPLES HOSP LIANYUNGANG | 2 | 0.197 |
| SHAANXI PROV PEOPLES HOSP | 2 | 0.197 |
| SHANDONG FIRST MED UNIV SHANDONG ACAD MED SCI | 2 | 0.197 |
| SHANGHAI GUANGHUA HOSP INTEGRATED TRADIT CHINESE | 2 | 0.197 |
| SHANXI ACAD MED SCI | 2 | 0.197 |
| SHAOXING UNIV | 2 | 0.197 |
| SHENZHEN LONGHUA DIST CENT HOSP | 2 | 0.197 |
| SHENZHEN PEOPLES HOSP | 2 | 0.197 |
| SICHUAN ACAD MED SCI | 2 | 0.197 |
| SOUTH CHINA AGR UNIV | 2 | 0.197 |
| SOUTHERN UNIV SCI TECHNOL | 2 | 0.197 |
| SOUTHWEST JIAOTONG UNIV | 2 | 0.197 |
| TAIZHOU PEOPLES HOSP | 2 | 0.197 |
| THIRD MIL MED UNIV | 2 | 0.197 |
| THIRD PEOPLES HOSP LINYI | 2 | 0.197 |
| TIANJIN MED UNIV CANC INST HOSP | 2 | 0.197 |
| TONGDE HOSP ZHEJIANG PROV | 2 | 0.197 |
| UNIV AMSTERDAM | 2 | 0.197 |
| UNIV CALIF LOS ANGELES | 2 | 0.197 |
| UNIV ELECT SCI TECHNOL CHINA | 2 | 0.197 |
| UNIV GIESSEN | 2 | 0.197 |
| UNIV LISBON | 2 | 0.197 |
| UNIV MILAN | 2 | 0.197 |
| UNIV SHANGHAI SCI TECHNOL | 2 | 0.197 |
| UNIV TORONTO | 2 | 0.197 |
| WRIGHT STATE UNIV | 2 | 0.197 |
| XIANGNAN UNIV | 2 | 0.197 |
| XINXIANG CENT HOSP | 2 | 0.197 |
| YANGTZE UNIV | 2 | 0.197 |
| YANGZHOU UNIV | 2 | 0.197 |
| YANTAISHAN HOSP | 2 | 0.197 |
| ZOUCHENG PEOPLES HOSP | 2 | 0.197 |
| 1 HOSP JILIN UNIV | 1 | 0.099 |
| 4 HOSP 1946 JINAN SHANDONG | 1 | 0.099 |
| 4 HOSP JINAN | 1 | 0.099 |
| 4TH CENT HOSP TIANJIN | 1 | 0.099 |
| 4TH XINYUAN HOSP YULIN | 1 | 0.099 |
| 5TH PEOPLES HOSP JINAN | 1 | 0.099 |
| 960TH HOSP CHINESE PLA | 1 | 0.099 |
| 988 HOSP JOINT LOGIST SUPPORT FORCE CHINESE PEOPL | 1 | 0.099 |
| AARUPADAI VEEDU MED COLL | 1 | 0.099 |
| AC CAMARGO CANC CTR | 1 | 0.099 |
| ACAD SINICA | 1 | 0.099 |
| AFFILIATED CANC HOSP ZHENGZHOU UNIV | 1 | 0.099 |
| AFFILIATED NANJING UNIV CHINESE MED | 1 | 0.099 |
| ALAGAPPA UNIV | 1 | 0.099 |
| ALBERT LUDWIGS UNIV FREIBURG | 1 | 0.099 |
| AMER UNIV BEIRUT | 1 | 0.099 |
| ANHUI 2 PROV PEOPLES HOSP | 1 | 0.099 |
| ANHUI ACAD TRADIT CHINESE MED | 1 | 0.099 |
| ANHUI CHEST HOSP | 1 | 0.099 |
| ANHUI GAOLU WINERY STAFF HOSP | 1 | 0.099 |
| ANHUI PROV KEY LAB HEPATOPANCREATOBILIARY SURG | 1 | 0.099 |
| ANHUI PROV KEY LAB MAJOR AUTOIMMUNE DIS | 1 | 0.099 |
| ANHUI PROV KEY LAB TRANSLAT CANC RES | 1 | 0.099 |
| ANHUI UNIV SCI TECHNOL | 1 | 0.099 |
| ANHUI UNIV TRADIT CHINESE M | 1 | 0.099 |
| ANHUI UNIV TRADIT CHINESE MED | 1 | 0.099 |
| ANIM DIS CONTROL CTR HAIXI MONGOLIAN TIBETAN AU | 1 | 0.099 |
| ANQING PETROCHEM HOSP | 1 | 0.099 |
| AO RES INST DAVOS | 1 | 0.099 |
| ARMY MED CTR PLA | 1 | 0.099 |
| ARMY MIL MED UNIV | 1 | 0.099 |
| ARS | 1 | 0.099 |
| BAOJI CTR HOSP SHANXI PROV | 1 | 0.099 |
| BAOTOU MED COLL | 1 | 0.099 |
| BARROW NEUROL INST | 1 | 0.099 |
| BEIJING INT SCI TECHNOL COOPERAT BASE TUMOR MET | 1 | 0.099 |
| BEIJING JIANLAN INST MED | 1 | 0.099 |
| BEIJING KEY LAB DRUG TARGETS IDENTIFICAT DRUG S | 1 | 0.099 |
| BEIJING UNIV CHEM TECHNOL | 1 | 0.099 |
| BEIJING UNIV CHINESE MED | 1 | 0.099 |
| BEIJING ZHONGKE JIANLAN BIOTECHNOL CO LTD | 1 | 0.099 |
| BERLIN INST HLTH | 1 | 0.099 |
| BINZHOU CENT HOSP | 1 | 0.099 |
| BINZHOU MED UNIV HOSP | 1 | 0.099 |
| BOXING PEOPLES HOSP | 1 | 0.099 |
| BUR ANIM HUSB BIYANG CTY | 1 | 0.099 |
| BUR ANIM HUSB HENAN PROV | 1 | 0.099 |
| CANGZHOU CENT HOSP HEBEI PROV | 1 | 0.099 |
| CARDIFF UNIV | 1 | 0.099 |
| CAROL DAVILA UNIV MED PHARM | 1 | 0.099 |
| CENT HOSP ENSHI TUJIA MIAO AUTONOMOUS PREFECTUR | 1 | 0.099 |
| CENT HOSP ZIBO | 1 | 0.099 |
| CENT PEOPLES HOSP ZHANJIANG | 1 | 0.099 |
| CGCG | 1 | 0.099 |
| CHANGHAI HOSP | 1 | 0.099 |
| CHANGLE CTY TANGWU TOWN HOSP | 1 | 0.099 |
| CHANGNING MATERN INFANT HLTH HOSP | 1 | 0.099 |
| CHANGZHOU CANC BIOMED TREATMENT CTR JIANGSU PROV | 1 | 0.099 |
| CHANGZHOU RD COMMUNITY HLTH SERV CTR HEDONG DIST | 1 | 0.099 |
| CHANGZHOU SEVENTH PEOPLES HOSP | 1 | 0.099 |
| CHARACTERIST MED CTR CHINESE PEOPLES ARMED POLICE | 1 | 0.099 |
| CHARITE | 1 | 0.099 |
| CHARITE MED FAC | 1 | 0.099 |
| CHENGDU MIL GEN HOSP | 1 | 0.099 |
| CHIFENG HOSP | 1 | 0.099 |
| CHIFENG TUMOR HOSP | 1 | 0.099 |
| CHILDRENS HOSP SHANXI PROV | 1 | 0.099 |
| CHINA AGR UNIV | 1 | 0.099 |
| CHINA COAST GUARD HOSP PEOPLES ARMED POLICE FORCE | 1 | 0.099 |
| CHINA JAPAN FRIENDSHIP HOSP | 1 | 0.099 |
| CHINA MED UNIV HOSP | 1 | 0.099 |
| CHINA ORTHOPED REGENERAT MED GRP CORMED | 1 | 0.099 |
| CHINA THREE GORGES UNIV | 1 | 0.099 |
| CHINESE CTR DIS CONTROL PREVENT | 1 | 0.099 |
| CHINESE CULTURE UNIV | 1 | 0.099 |
| CHINESE PEOPLES LIBERAT ARMY | 1 | 0.099 |
| CHINESE PLA GEN HOSP BEIJING | 1 | 0.099 |
| CHONGQING CANC HOSP | 1 | 0.099 |
| CHONGQING CTR DRUG CERTIFICAT EVALUAT | 1 | 0.099 |
| CHONGQING HOSP TRADIT CHINESE MED | 1 | 0.099 |
| CHONGQING THREE GORGES MED COLL | 1 | 0.099 |
| CHONGQING THREE GORGES UNIV | 1 | 0.099 |
| CHONGQING TRADIT CHINESE MED HOSP | 1 | 0.099 |
| COLLABORAT INNOVAT CTR BIOTHERAPY | 1 | 0.099 |
| COLLABORAT INNOVAT CTR CANC MED | 1 | 0.099 |
| COMMUNITY HLTH SERV CTR | 1 | 0.099 |
| CSIC | 1 | 0.099 |
| CTR PAIN RES TREATMENT | 1 | 0.099 |
| DALIAN JIAOTONG UNIV | 1 | 0.099 |
| DARTMOUTH COLL | 1 | 0.099 |
| DEPT MED ONCOL | 1 | 0.099 |
| DEPT SCI TECHNOL GUANGDONG PROV | 1 | 0.099 |
| DIV GASTROINTESTINAL SURG | 1 | 0.099 |
| DONGGUAN WATERFRONT ZONE CENT HOSP | 1 | 0.099 |
| DONGYING DIST HOSP | 1 | 0.099 |
| DONGYING SHENGLI HOSP | 1 | 0.099 |
| DR SENCKENBERG INST PATHOL | 1 | 0.099 |
| DRUG REHABIL CTR HEILONGJIANG PROV | 1 | 0.099 |
| DUKE UNIV | 1 | 0.099 |
| ENGN RES CTR MOL DIAG CELL TREATMENT METAB BONE | 1 | 0.099 |
| FASA UNIV MED SCI | 1 | 0.099 |
| FDN IRCCS CA GRANDA OSPED MAGGIORE POLICLIN | 1 | 0.099 |
| FDN SCI TECHNOL | 1 | 0.099 |
| FIFTH HOSP HARBIN | 1 | 0.099 |
| FIFTH HOSP WUHAN | 1 | 0.099 |
| FIFTH PEOPLES HOSP JINAN CITY | 1 | 0.099 |
| FIFTH PEOPLES HOSP SHANGHAI | 1 | 0.099 |
| FIRST AFFILIATED HOSP HARBIN MED UNIV | 1 | 0.099 |
| FIRST AFFILIATED HOSP HEBEI NORTH | 1 | 0.099 |
| FIRST AFFILIATED YIJISHAN HOSP | 1 | 0.099 |
| FIRST HOSP SUIHUA | 1 | 0.099 |
| FIRST HOSP YULIN | 1 | 0.099 |
| FIRST HOSP ZIBO | 1 | 0.099 |
| FIRST MED UNIV | 1 | 0.099 |
| FIRST PEOPLES HOSP HONGHE STATE | 1 | 0.099 |
| FIRST PEOPLES HOSP KUNSHAN | 1 | 0.099 |
| FIRST PEOPLES HOSP LIANYUNGANG CITY | 1 | 0.099 |
| FIRST PEOPLES HOSP QUJING | 1 | 0.099 |
| FIRST PEOPLES HOSP SHANGQIU | 1 | 0.099 |
| FLORIDA STATE UNIV | 1 | 0.099 |
| FOSHAN UNIV | 1 | 0.099 |
| FOURTH PEOPLES HOSP SHAANXI | 1 | 0.099 |
| FOURTH PEOPLES HOSP SHENYANG | 1 | 0.099 |
| FUJIAN KEY LAB TRANSLAT CANC MED | 1 | 0.099 |
| FUJIAN MED SCI RES INST | 1 | 0.099 |
| FUJIAN MED UNIV UNION HOSP | 1 | 0.099 |
| FUJIAN PROV CANC HOSP | 1 | 0.099 |
| FUJIAN PROV KEY LAB TUMOR BIOTHERAPY | 1 | 0.099 |
| FUJIAN UNIV TRADIT CHINESE MED | 1 | 0.099 |
| FUNDACAO ANTONIO PRUDENTE | 1 | 0.099 |
| FUZHOU CTR DIS CONTROL PREVENT | 1 | 0.099 |
| FUZHOU UNIV | 1 | 0.099 |
| GANSU PROV PEOPLES HOSP | 1 | 0.099 |
| GAOMI PEOPLES HOSP | 1 | 0.099 |
| GEN HOSP JINAN MIL COMMAND | 1 | 0.099 |
| GEN HOSP NORTHERN THEATER COMMAND | 1 | 0.099 |
| GENOME INST SINGAPORE | 1 | 0.099 |
| GOETHE UNIV | 1 | 0.099 |
| GONGLI HOSP PUDONG NEW AREA | 1 | 0.099 |
| GUANGDONG HIGHER EDUC INST GUANGDONG PROV | 1 | 0.099 |
| GUANGDONG INST APPL BIOL RESOURCES | 1 | 0.099 |
| GUANGDONG KEY LAB ORTHOPAED TECHNOL IMPLANT MAT | 1 | 0.099 |
| GUANGDONG KEY LAB STOMATOL | 1 | 0.099 |
| GUANGDONG LAB ANIM MONITORING INST | 1 | 0.099 |
| GUANGDONG LEWWIN PHARMACEUT RES INST CO LTD | 1 | 0.099 |
| GUANGDONG OCEAN UNIV | 1 | 0.099 |
| GUANGDONG PROV ENGN LAB TRANSPLANTAT MED | 1 | 0.099 |
| GUANGDONG PROV FAMILY PLANNING SCI TECHNOL RES | 1 | 0.099 |
| GUANGDONG PROV KEY LAB MOL TUMOR PATHOL | 1 | 0.099 |
| GUANGDONG SECOND PROV GEN HOSP | 1 | 0.099 |
| GUANGDONG WOMEN CHILDREN HOSP | 1 | 0.099 |
| GUANGXI CLIN RES CTR CARDIOCEREBROVASC DIS | 1 | 0.099 |
| GUANGXI KEY LAB BASE PRECIS MED CARDIOCEREBROVASC | 1 | 0.099 |
| GUANGZHOU MIL COMMAND | 1 | 0.099 |
| GUANGZHOU REGENERAT MED HLTH GUANGDONG LAB | 1 | 0.099 |
| GUILIN MED COLL | 1 | 0.099 |
| GUIYANG MATERNAL CHILD HLTH HOSP | 1 | 0.099 |
| GULLIVER PREPARATORY SCH | 1 | 0.099 |
| GUSHANG HOSP GUANGXI ZHUANG AUTONOMOUS REG | 1 | 0.099 |
| HAINAN ACAD AGR SCI | 1 | 0.099 |
| HAINAN GEN HOSP | 1 | 0.099 |
| HAINAN MED UNIV | 1 | 0.099 |
| HANGZHOU CANC HOSP | 1 | 0.099 |
| HANGZHOU HOSP | 1 | 0.099 |
| HANGZHOU THIRD HOSP | 1 | 0.099 |
| HANGZHOU THIRD PEOPLES HOSP | 1 | 0.099 |
| HANZHONG CENT HOSP | 1 | 0.099 |
| HARBIN ENGN UNIV | 1 | 0.099 |
| HARBIN MED UNIV CANC HOSP | 1 | 0.099 |
| HARBIN PHARMACEUT GRP | 1 | 0.099 |
| HARBIN STOMATOL HOSP | 1 | 0.099 |
| HEBEI NORTH UNIV | 1 | 0.099 |
| HEBEI UNIV | 1 | 0.099 |
| HEBEI UNIV CHINESE MED | 1 | 0.099 |
| HEBEI UNIV ENGN | 1 | 0.099 |
| HEILONGJIANG ACAD TRADIT CHINESE MED | 1 | 0.099 |
| HEILONGJIANG AGR RECLAMAT BUR | 1 | 0.099 |
| HEILONGJIANG UNIV CHINESE MED | 1 | 0.099 |
| HENAN AGR UNIV | 1 | 0.099 |
| HENAN CHEST HOSP | 1 | 0.099 |
| HENAN ENGN RES CTR CLIN DATA BIOBANK CARDIOVASC | 1 | 0.099 |
| HENAN MED KEY LAB MOL IMAGING | 1 | 0.099 |
| HENAN PROV CHEST HOSP | 1 | 0.099 |
| HENAN TRADIT CHINESE MED HOSP | 1 | 0.099 |
| HENAN UNIV CHINESE MED | 1 | 0.099 |
| HENAN UNIV PEOPLES HOSP | 1 | 0.099 |
| HENAN UNIV TRADIT CHINESE MED | 1 | 0.099 |
| HEZE INFECT DIS HOSP | 1 | 0.099 |
| HIROSHIMA UNIV | 1 | 0.099 |
| HLTH TIME GENE INST | 1 | 0.099 |
| HONG KONG UNIV SCI TECHNOL | 1 | 0.099 |
| HOSP UNIV VALME | 1 | 0.099 |
| HUANGHE CENT HOSP | 1 | 0.099 |
| HUAZHONG UNIV SCI TECHNOL HUST | 1 | 0.099 |
| HUBEI CANC HOSP | 1 | 0.099 |
| HUBEI KEY LAB KIDNEY DIS PATHOGENESIS INTERVENT | 1 | 0.099 |
| HUBEI UNIV ARTS SCI | 1 | 0.099 |
| HUBEI UNIV CHINESE MED | 1 | 0.099 |
| HUBEI UNIV SCI TECHNOL | 1 | 0.099 |
| HUIZHOU MUNICIPAL CENT HOSP | 1 | 0.099 |
| HUMANITAS RES HOSP | 1 | 0.099 |
| HUMANITAS UNIV | 1 | 0.099 |
| HUNAN CANC HOSP | 1 | 0.099 |
| HUNAN CLIN RES CTR OPHTHALM DIS | 1 | 0.099 |
| HUNAN KEY LAB OPHTHALMOL | 1 | 0.099 |
| HUNAN NORMAL UNIV | 1 | 0.099 |
| HUNAN PROV KEY LAB TUMOR CELLULAR MOL PATHOL 20 | 1 | 0.099 |
| HUNTER MED RES INST | 1 | 0.099 |
| ICAHN SCH MED MT SINAI | 1 | 0.099 |
| INNER MONGOLIA KEY LAB MONGOLIAN MED PHARMACOL CA | 1 | 0.099 |
| INNER MONGOLIA MED UNIV | 1 | 0.099 |
| INNER MONGOLIA UNIV NATIONALITIES | 1 | 0.099 |
| INST CARDIOVASC REGENERAT | 1 | 0.099 |
| INST LIFE SCI | 1 | 0.099 |
| INT BREAST CANC NUTR IBCN PROJECT | 1 | 0.099 |
| IRCCS REGINA ELENA NATL CANC INST | 1 | 0.099 |
| IST NAZL GENET MOL ROMEO ENRICA INVERNIZZI | 1 | 0.099 |
| IST NAZL TUMORI IRCCS FDN G PASCALE | 1 | 0.099 |
| IULIU HATIEGANU UNIV MED PHARM | 1 | 0.099 |
| JAGIELLONIAN UNIV | 1 | 0.099 |
| JIAN HOSP | 1 | 0.099 |
| JIANGDU PEOPLES HOSP YANGZHOU | 1 | 0.099 |
| JIANGMEN CENT HOSP | 1 | 0.099 |
| JIANGSU COLL NURSING | 1 | 0.099 |
| JIANGSU ENGN RES CTR TUMOR IMMUNOTHERAPY | 1 | 0.099 |
| JIANGSU HENGRUI MED | 1 | 0.099 |
| JIANGSU NORMAL UNIV | 1 | 0.099 |
| JIANGSU PROV CORPS HOSP CHINESE PEOPLES ARMED POL | 1 | 0.099 |
| JIANGSU PROV HOSP CHINESE MED | 1 | 0.099 |
| JIANGSU TAIZHOU PEOPLES HOSP | 1 | 0.099 |
| JIANGXI CANC HOSP | 1 | 0.099 |
| JIANGXI CHEST HOSP | 1 | 0.099 |
| JIANGXI MATERNAL CHILD HLTH HOSP | 1 | 0.099 |
| JIANGXI PROV MATERNAL CHILD HLTH HOSP | 1 | 0.099 |
| JIAOTONG UNIV | 1 | 0.099 |
| JIAOZUO PEOPLES HOSP | 1 | 0.099 |
| JILIN CANC HOSP | 1 | 0.099 |
| JILIN MED UNIV | 1 | 0.099 |
| JILIN PROV BLOOD CTR | 1 | 0.099 |
| JINAN CENT HOSP | 1 | 0.099 |
| JINAN CITY PEOPLES HOSP | 1 | 0.099 |
| JINAN MATERN CHILD CARE HOSP | 1 | 0.099 |
| JINGLING HOSP | 1 | 0.099 |
| JINING HOSP TCM | 1 | 0.099 |
| JINING MATERNAL CHILD HLTH FAMILY PLANNING SERV | 1 | 0.099 |
| JINING NO 1 PEOPLES HOSP | 1 | 0.099 |
| KARADENIZ TECH UNIV | 1 | 0.099 |
| KEY LAB DIAG TREATMENT DIGEST SYST TUMORS ZHEJI | 1 | 0.099 |
| KEY LAB NEUROONCOL LIAONING PROV | 1 | 0.099 |
| KOREA RES INST BIOSCI BIOTECHNOL KRIBB | 1 | 0.099 |
| KUNMING ANGEL WOMENS CHILDRENS HOSP | 1 | 0.099 |
| KURUME UNIV | 1 | 0.099 |
| KYUNGPOOK NATL UNIV | 1 | 0.099 |
| LAIZHOU CITY PEOPLES HOSP | 1 | 0.099 |
| LANZHOU UNIV SECOND HOSP | 1 | 0.099 |
| LIANYUNGANG MUNICIPAL ORIENTAL HOSP | 1 | 0.099 |
| LIANYUNGANG SECOND PEOPLES HOSP | 1 | 0.099 |
| LIAONING PROV CANC HOSP | 1 | 0.099 |
| LIAONING RES CTR TRANSLAT MED NERVOUS SYST DIS | 1 | 0.099 |
| LIAONING UNIV TRADIT CHINESE MED | 1 | 0.099 |
| LINYI CANC HOSP | 1 | 0.099 |
| LISBON UNIV | 1 | 0.099 |
| LISHUI UNIV | 1 | 0.099 |
| LIUZHOU PEOPLES HOSP | 1 | 0.099 |
| LIYANG PEOPLES HOSP | 1 | 0.099 |
| LONGYAN UNIV | 1 | 0.099 |
| LOUISIANA STATE UNIV | 1 | 0.099 |
| MAASTRICHT UNIV | 1 | 0.099 |
| MASSACHUSETTS GEN HOSP | 1 | 0.099 |
| MASSACHUSETTS UNIV | 1 | 0.099 |
| MATERNAL CHILD HLTH HOSP HUBEI PROV | 1 | 0.099 |
| MATERNAL CHILD HLTH HOSP HUNAN | 1 | 0.099 |
| MAX DELBRUCK CTR MOL MED | 1 | 0.099 |
| MED CTR ASSESSMENT PREVENT TREATMENT BONE JOI | 1 | 0.099 |
| MED REFORM MANAGEMENT OFF | 1 | 0.099 |
| MEDAXIS TECHNOL CO LTD | 1 | 0.099 |
| MEM SLOAN KETTERING CANC CTR | 1 | 0.099 |
| MICHIGAN STATE UNIV | 1 | 0.099 |
| MINIST AGR CHINA | 1 | 0.099 |
| MUDANJIANG MED COLL | 1 | 0.099 |
| NANFANG HOSP | 1 | 0.099 |
| NANJING DRUM TOWER HOSP | 1 | 0.099 |
| NANJING TRADIT CHINESE MED HOSP | 1 | 0.099 |
| NANJING TRADIT CHINESE MED HOSP LIUHE DIST | 1 | 0.099 |
| NANJING UNIV AERONUT ASTRONAUT | 1 | 0.099 |
| NANJING UNIV POSTS TELECOMMUN | 1 | 0.099 |
| NANKAI UNIV | 1 | 0.099 |
| NANTONG SECOND PEOPLES HOSP | 1 | 0.099 |
| NANTONG TUMOR HOSP | 1 | 0.099 |
| NATL CLIN RES CTR DIGEST DIS | 1 | 0.099 |
| NATL CLIN RES CTR ORAL DIS | 1 | 0.099 |
| NATL ENGN LAB ORAL REGENERAT MED | 1 | 0.099 |
| NATL LOCAL JOINT ENGN RES CTR LIVESTOCK BREEDING | 1 | 0.099 |
| NATL TAIWAN UNIV | 1 | 0.099 |
| NATL TAIWAN UNIV HOSP | 1 | 0.099 |
| NATL YANG MING UNIV | 1 | 0.099 |
| NEUROSCI RES AUSTRALIA | 1 | 0.099 |
| NEW DIST LONGHUA SHENZHEN | 1 | 0.099 |
| NIA | 1 | 0.099 |
| NINGBO CLIN PATHOL DIAG CTR | 1 | 0.099 |
| NINGBO CLIN RES CTR DIGEST SYST TUMORS | 1 | 0.099 |
| NINGBO HANGZHOU BAY HOSP | 1 | 0.099 |
| NINGBO UROL NEPHROL HOSP | 1 | 0.099 |
| NINGBO WOMEN CHILDRENS HOSP | 1 | 0.099 |
| NINGBO YINZHOU 2 HOSP | 1 | 0.099 |
| NINGHAI FIRST HOSP | 1 | 0.099 |
| NINGHAI HOSP | 1 | 0.099 |
| NORTH SICHUAN MED COLL | 1 | 0.099 |
| NORTHERN JIANGSU PEOPLES HOSP | 1 | 0.099 |
| NORWEGIAN UNIV SCI TECHNOL | 1 | 0.099 |
| NUST | 1 | 0.099 |
| NYU | 1 | 0.099 |
| OKAYAMA UNIV | 1 | 0.099 |
| ONCOL HOSP JIAMUSI CITY | 1 | 0.099 |
| PAYAME NOOR UNIV | 1 | 0.099 |
| PEKING UNION MED COLL CHINESE ACAD MED SCI | 1 | 0.099 |
| PEKING UNION MED COLL HOSP | 1 | 0.099 |
| PEKING UNIV SHENZHEN HOSP | 1 | 0.099 |
| PENGLAI PEOPLES HOSP | 1 | 0.099 |
| PENN STATE UNIV | 1 | 0.099 |
| PEOPLES HOSP BINZHOU | 1 | 0.099 |
| PEOPLES HOSP CHINA THREE GORGES UNIV | 1 | 0.099 |
| PEOPLES HOSP GANZHOU | 1 | 0.099 |
| PEOPLES HOSP GAOTANG | 1 | 0.099 |
| PEOPLES HOSP GAOZHOU | 1 | 0.099 |
| PEOPLES HOSP GUAZHOU CTY | 1 | 0.099 |
| PEOPLES HOSP HENAN PROV | 1 | 0.099 |
| PEOPLES HOSP HONGZE DIST | 1 | 0.099 |
| PEOPLES HOSP HUANGYUAN CTY | 1 | 0.099 |
| PEOPLES HOSP JIAOZUO | 1 | 0.099 |
| PEOPLES HOSP LANLING CTY | 1 | 0.099 |
| PEOPLES HOSP LINSHU | 1 | 0.099 |
| PEOPLES HOSP TONGCHUAN | 1 | 0.099 |
| PEOPLES LIBERAT ARMY | 1 | 0.099 |
| PEOPLES LIBERAT ARMY GEN HOSP | 1 | 0.099 |
| PINGYI HOSP TRADIT CHINESE MED | 1 | 0.099 |
| PLA | 1 | 0.099 |
| PLA 904 HOSP | 1 | 0.099 |
| PLA NAVY ANQING HOSP | 1 | 0.099 |
| PLA ROCKET FORCE CHARACTERIST MED CTR | 1 | 0.099 |
| POLISH ACAD SCI | 1 | 0.099 |
| PROV KEY LAB RESP DIS ANHUI | 1 | 0.099 |
| PUSAN NATL UNIV | 1 | 0.099 |
| QINGDAO HLTH SCH SHANDONG PROV | 1 | 0.099 |
| QINGDAO NATL LAB MARINE SCI TECHNOL | 1 | 0.099 |
| QINHUANGDAO HAIGANG HOSP | 1 | 0.099 |
| QIONGTAI NORMAL UNIV | 1 | 0.099 |
| RIZHAO CITY TRADIT CHINESE MED HOSP | 1 | 0.099 |
| ROYAL INFIRM EDINBURGH NHS TRUST | 1 | 0.099 |
| ROYAL VICTORIAN EYE EAR HOSP | 1 | 0.099 |
| RUSSIAN ACAD MED SCI | 1 | 0.099 |
| SAARLAND UNIV | 1 | 0.099 |
| SCH MED KAIFENG CITY | 1 | 0.099 |
| SECOND HOSP LIANYUNGANG | 1 | 0.099 |
| SECOND HOSP WEIFANG | 1 | 0.099 |
| SECOND PEOPLES HOSP HEFEI | 1 | 0.099 |
| SECOND PEOPLES HOSP PINGYANG CTY | 1 | 0.099 |
| SECOND PEOPLES HOSP SHENZHEN | 1 | 0.099 |
| SECOND PEOPLES HOSP WUHU | 1 | 0.099 |
| SECONDARY MIL MED UNIV | 1 | 0.099 |
| SHAANXI PEOPLES HOSP | 1 | 0.099 |
| SHAANXI PROV TUMOR HOSP | 1 | 0.099 |
| SHANDONG ACAD CHINESE MED | 1 | 0.099 |
| SHANDONG ENGN LAB DENT MAT ORAL TISSUE REGENERA | 1 | 0.099 |
| SHANDONG EYE INST | 1 | 0.099 |
| SHANDONG KEY LAB ORAL TISSUE REGENERAT | 1 | 0.099 |
| SHANDONG LUNAN EYE HOSP | 1 | 0.099 |
| SHANDONG MED COLL | 1 | 0.099 |
| SHANDONG PROV COAL TAISHAN SANAT | 1 | 0.099 |
| SHANDONG PROV THIRD HOSP | 1 | 0.099 |
| SHANDONG SHANXIAN CENT HOSP | 1 | 0.099 |
| SHANDONG SUNSHINE UNION HOSP CO LTD | 1 | 0.099 |
| SHANGHAI EIGHTH PEOPLE HOSP | 1 | 0.099 |
| SHANGHAI INST DERMATOL | 1 | 0.099 |
| SHANGHAI INST DIGEST DIS | 1 | 0.099 |
| SHANGHAI INST PEDIAT RES | 1 | 0.099 |
| SHANGHAI JIAO TONG UNIV MED | 1 | 0.099 |
| SHANGHAI KEY LAB BILIARY TRACT DIS RES | 1 | 0.099 |
| SHANGHAI MUNICIPAL CTR DIS CONTROL PREVENT | 1 | 0.099 |
| SHANGHAI OCEAN UNIV | 1 | 0.099 |
| SHANGHAI PUDONG NEW AREA GONGLI HOSP | 1 | 0.099 |
| SHANGHAI RES INST STOMATOL | 1 | 0.099 |
| SHANGHAI UNIV | 1 | 0.099 |
| SHANGHAI UNIV SPORT | 1 | 0.099 |
| SHANGQIU FIRST PEOPLES HOSP | 1 | 0.099 |
| SHANTOU CENT HOSP | 1 | 0.099 |
| SHANTOU UNIV MED COLL | 1 | 0.099 |
| SHANXI AGR UNIV | 1 | 0.099 |
| SHANXI BETHUNE HOSP | 1 | 0.099 |
| SHANXI EYE HOSP | 1 | 0.099 |
| SHANXI HUAJIN ORTHOPED HOSP | 1 | 0.099 |
| SHAOXING SHANGYU PEOPLES HOSP | 1 | 0.099 |
| SHAPINGBA PEOPLES HOSP | 1 | 0.099 |
| SHENYANG MED COLL | 1 | 0.099 |
| SHENYANG PHARMACEUT UNIV | 1 | 0.099 |
| SHENZHEN BAOAN DIST SONGGANG PEOPLES HOSP | 1 | 0.099 |
| SHENZHEN COLL INT EDUC | 1 | 0.099 |
| SHENZHEN FUTIAN HOSP RHEUMAT DIS | 1 | 0.099 |
| SHENZHEN PEKING UNIV HONG KONG UNIV SCI TECHNOL | 1 | 0.099 |
| SHIDONG HOSP | 1 | 0.099 |
| SHOUGUANG PEOPLE HOSP | 1 | 0.099 |
| SHOUYI MARIA MATERN HOSP | 1 | 0.099 |
| SICHUAN AGR UNIV | 1 | 0.099 |
| SICHUAN PROV PEOPLES HOSP | 1 | 0.099 |
| SISHUI PEOPLES HOSP | 1 | 0.099 |
| SIVAS CUMHURIYET UNIV | 1 | 0.099 |
| SOUTH CHINA COLLABORAT INNOVAT CTR POULTRY DIS CO | 1 | 0.099 |
| SOUTH CHINA UNIV TECHNOL | 1 | 0.099 |
| SOUTHERN MED UNIV DONGGUAN | 1 | 0.099 |
| SOUTHWEST UNIV | 1 | 0.099 |
| SOUTHWEST UNIV NATIONALITIES | 1 | 0.099 |
| SPIN LTDA EPP | 1 | 0.099 |
| ST VINCENTS HOSP | 1 | 0.099 |
| STANFORD CARDIOVASC INST | 1 | 0.099 |
| STATE KEY LAB CULTIVAT BASE CELL DIFFERENTIAT REG | 1 | 0.099 |
| STATE KEY LAB ONCOL SOUTHERN CHINA | 1 | 0.099 |
| STATE PROV KEY LABS BIOMED PHARMACEUT CHINA | 1 | 0.099 |
| STICHTING EPILEPSIE INSTELLINGEN NEDERLAND | 1 | 0.099 |
| SUINING CENT HOSP | 1 | 0.099 |
| SUN YAT SEN MEM HOSP | 1 | 0.099 |
| SUNNYBROOK HLTH SCI CTR | 1 | 0.099 |
| SUQIAN FIRST HOSP | 1 | 0.099 |
| SUZHOU SCI TECHNOL TOWN HOSP | 1 | 0.099 |
| TAIAN CITY CENT HOSP | 1 | 0.099 |
| TAIPEI VET GEN HOSP | 1 | 0.099 |
| TAIXING PEOPLES HOSP | 1 | 0.099 |
| TAIYUAN IRON STEEL GRP | 1 | 0.099 |
| TAIZHOU HOSP ZHEJIANG PROV | 1 | 0.099 |
| TAIZHOU TRADIT CHINESE MED HOSP | 1 | 0.099 |
| TAIZHOU UNIV HOSP | 1 | 0.099 |
| TANGSHAN WORKERS HOSP | 1 | 0.099 |
| TENGZHOU CENT PEOPLES HOSP | 1 | 0.099 |
| THIRD CENT HOSP TIANJIN | 1 | 0.099 |
| THIRD HOSP JINAN | 1 | 0.099 |
| THIRD PEOPLES HOSP CHENGDU | 1 | 0.099 |
| THIRD PEOPLES HOSP DALIAN | 1 | 0.099 |
| TIANJIN FIRST CTR HOSP | 1 | 0.099 |
| TIANJIN MED COLL | 1 | 0.099 |
| TIANJIN UNIV TRADIT CHINESE MED | 1 | 0.099 |
| TIANJING MED UNIV | 1 | 0.099 |
| TOHOKU UNIV | 1 | 0.099 |
| TONGLING PEOPLES HOSP | 1 | 0.099 |
| TONGREN SECOND PEOPLES HOSP | 1 | 0.099 |
| TUMOR HOSP SHANXI | 1 | 0.099 |
| TUMOR HOSP SHANXI PROV | 1 | 0.099 |
| TUMOR HOSP YUNNAN PROV | 1 | 0.099 |
| TUMOUR HOSP LIAOCHENG | 1 | 0.099 |
| UCL | 1 | 0.099 |
| UCLA | 1 | 0.099 |
| UNIV ALABAMA BIRMINGHAM | 1 | 0.099 |
| UNIV BRESCIA | 1 | 0.099 |
| UNIV CALGARY | 1 | 0.099 |
| UNIV EDINBURGH | 1 | 0.099 |
| UNIV EXETER | 1 | 0.099 |
| UNIV FED RIO GRANDE DO SUL | 1 | 0.099 |
| UNIV GRONINGEN | 1 | 0.099 |
| UNIV HAWAII MANOA | 1 | 0.099 |
| UNIV HONG KONG | 1 | 0.099 |
| UNIV LEIPZIG | 1 | 0.099 |
| UNIV MELBOURNE | 1 | 0.099 |
| UNIV MIAMI | 1 | 0.099 |
| UNIV MICHIGAN | 1 | 0.099 |
| UNIV MISSISSIPPI | 1 | 0.099 |
| UNIV MODENA REGGIO EMILIA | 1 | 0.099 |
| UNIV NAPLES FEDERICO II | 1 | 0.099 |
| UNIV NEW SOUTH WALES | 1 | 0.099 |
| UNIV NEW SOUTH WALES SYDNEY | 1 | 0.099 |
| UNIV NEWCASTLE | 1 | 0.099 |
| UNIV NYALA | 1 | 0.099 |
| UNIV PENN | 1 | 0.099 |
| UNIV PITTSBURGH | 1 | 0.099 |
| UNIV PLYMOUTH | 1 | 0.099 |
| UNIV SAO PAULO | 1 | 0.099 |
| UNIV SEVILLE | 1 | 0.099 |
| UNIV TEXAS MD ANDERSON CANC CTR | 1 | 0.099 |
| UNIV TEXAS MED BRANCH | 1 | 0.099 |
| UNIV TOYAMA | 1 | 0.099 |
| UNIV TUNKU ABDUL RAHMAN | 1 | 0.099 |
| UNIV VIRGINIA | 1 | 0.099 |
| UPSTATE MED UNIV | 1 | 0.099 |
| VIRGINIA COMMONWEALTH UNIV | 1 | 0.099 |
| VNJE UNIV AMSTERDAM | 1 | 0.099 |
| VRYE UNIV AMSTERDAM | 1 | 0.099 |
| WANBEI COAL ELECT GRP GEN HOSP | 1 | 0.099 |
| WEI FANG TRADIT CHINESE HOSP | 1 | 0.099 |
| WEIFANG MATERNAL CHILD HLTH HOSP | 1 | 0.099 |
| WEIFANG MENTAL HLTH CTR | 1 | 0.099 |
| WEIFANG YIDU CTR HOSP | 1 | 0.099 |
| WEIHAI STOMATOL HOSP | 1 | 0.099 |
| WEIZMANN INST SCI | 1 | 0.099 |
| WENLING FIRST PEOPLES HOSP | 1 | 0.099 |
| WENZHOU CENT HOSP | 1 | 0.099 |
| WENZHOU PEOPLES HOSP | 1 | 0.099 |
| WILLIAM BEAUMONT HOSP | 1 | 0.099 |
| WOMEN CHILDREN HLTH INST FUTIAN SHENZHEN | 1 | 0.099 |
| WUHAN ASIA GEN HOSP | 1 | 0.099 |
| WUHAN CENT HOSP | 1 | 0.099 |
| WUHAN TONGJI REPROD MED HOSP | 1 | 0.099 |
| XIAMEN MUNICIPAL KEY LAB GASTROINTESTINAL ONCOL | 1 | 0.099 |
| XIAN 3 HOSP | 1 | 0.099 |
| XIAN FOURTH HOSP | 1 | 0.099 |
| XIAN MED COLL | 1 | 0.099 |
| XIAOSHAN HOSP | 1 | 0.099 |
| XINXIANG MED COLL | 1 | 0.099 |
| XINYANG NORMAL UNIV | 1 | 0.099 |
| XIXI HOSP HANGZHOU | 1 | 0.099 |
| XUANWU LAKE COMMUNITY HLTH SERV CTR | 1 | 0.099 |
| XUHUI CENT HOSP | 1 | 0.099 |
| YALE UNIV | 1 | 0.099 |
| YAN DA INT HOSP | 1 | 0.099 |
| YANAN UNIV | 1 | 0.099 |
| YANCHENG CITY 1 PEOPLES HOSP | 1 | 0.099 |
| YANTAI YEDA HOSP | 1 | 0.099 |
| YIDU CENT HOSP WEIFANG | 1 | 0.099 |
| YINCHUAN FIRST PEOPLES HOSP | 1 | 0.099 |
| YOUJIANG MED UNIV NAT | 1 | 0.099 |
| YOUJIANG MED UNIV NATIONALITES | 1 | 0.099 |
| YOUJIANG MED UNIV NATIONALITIES | 1 | 0.099 |
| YUNNAN ACAD GRASSLAND ANIM SCI | 1 | 0.099 |
| YUNNAN PROV HOSP TRADIT CHINESE MED | 1 | 0.099 |
| ZHANGZHOU HLTH VOCAT COLL | 1 | 0.099 |
| ZHEJIANG INTEGRATED WESTERN MED HOSP | 1 | 0.099 |
| ZHEJIANG PROV KEY LAB OPHTHALMOL | 1 | 0.099 |
| ZHENGXING HOSP | 1 | 0.099 |
| ZHENGZHOU HOSP TRADIT CHINESE MED | 1 | 0.099 |
| ZHENGZHOU PEOPLES HOSP | 1 | 0.099 |
| ZHENGZHOU YIHE HOSP | 1 | 0.099 |
| ZHONGKAI UNIV AGR ENGN | 1 | 0.099 |
| ZHONGSHAN PEOPLES HOSP | 1 | 0.099 |
| ZHOUKOU CENT HOSP | 1 | 0.099 |
| ZHUJI CENT HOSP ZHEJIANG PROV | 1 | 0.099 |
| ZHUJI PEOPLES HOSP ZHEJIANG PROV | 1 | 0.099 |
| ZHUZHOU CENT HOSP | 1 | 0.099 |
| ZIBO CENT HOSP | 1 | 0.099 |
| ZIBO MATERNAL CHILD HLTH HOSP | 1 | 0.099 |
| ZUNYI MED UNIV | 1 | 0.099 |
